# Nonunion Humerous Fracture Infection Caused by *Rhizobium radiobacter* in a 24-Year-Old Healthy Patient: A Rare Case Report

**DOI:** 10.1155/2018/8627165

**Published:** 2018-08-12

**Authors:** Aikaterini Stamou, Charalampos Pavlopoulos, Stefanos Roumeliotis, Efthymios Samoladas, Ippokratis Xatzokos, Konstantina Kontopoulou

**Affiliations:** ^1^Laboratory of Microbiology, “G. Gennimatas” General Hospital Thessaloniki, Thessaloniki, Greece; ^2^2nd Orthopaedic Department, Aristotle University of Thessaloniki, Thessaloniki, Greece; ^3^2nd Orthopaedic Department, “G. Gennimatas” General Hospital Thessaloniki, Thessaloniki, Greece; ^4^Division of Nephrology and Hypertension, 1st Department of Internal Medicine, AHEPA Hospital, School of Medicine, Aristotle University of Thessaloniki, Thessaloniki, Greece

## Abstract

Although *Rhizobium radiobacter* is a pathogen commonly found in soil and plants, human disease caused by the *Rhizobium* genus is rare and cited in immunocompromised patients and in those who carry foreign plastic bodies such as catheters. We present a case of a 24-year-old woman with an adequate immune system who underwent surgery for an open fracture of the right tibia and humerus due to a car accident. One year later, she was readmitted to the hospital, due to a nonunion of the humeral fracture for surgical debridement and revision of the internal fixation with iliac crest autograft. *Rhizobium radiobacter* was isolated from the nonunion site, and the patient was treated with intramuscular administration of amikacin for 3 weeks followed by doxycycline per os for 8 weeks. After 3 months, the patient showed complete remission of the infection, substantial improvement, and union on the X-ray images. This is the first case of *Rhizobium radiobacter* infection in a patient with an adequate immune system that did not carry any foreign body and probably was initially infected due to open wound exposure to soil. Treatment of *R. radiobacter* infections should be individualised according to the antimicrobial susceptibility test for a successful infection management.

## 1. Introduction

*Rhizobium radiobacter* was formerly known as *Agrobacterium radiobacter* until recently, when *Agrobacterium* spp. were reclassified based on comparative 16S rRNA gene analyses. It is an aerobic, motile, oxidase-positive, non-spore-forming Gram-negative phytopathogenic bacillus that resembles Centers for Disease Control (CDC) group Vd-3 [1, 2]. Among the 5 species of the genus *Rhizobium, R. radiobacter* is the only one known to cause human disease, though it has a low virulence for humans [3, 4]. It is a rare opportunistic organism in human infections, which was never reported isolated in infected nonunions. Before 1977, the growth of *Rhizobium* species was mostly considered as laboratory contaminant or colonization rather than true infection [5]. The first case of human infection due to *R. radiobacter* was recorded in 1980 concerning an episode of endocarditis related to the prosthetic aortic valve [6]. Since then and during the last thirty eight years, an increasing number of infections associated with *R. radiobacter* have been reported in humans, especially immunocompromised or carriers of long-standing indwelling foreign devices or plastic materials, with which these organisms have marked adhesion capacity [7, 8].

Based on the limited knowledge of *R. radiobacter,* infected nonunions by *R. radiobacter* are never reported. Herein, we report one case of infected nonunion caused by *R. radiobacter* in a patient with an intact immune system after a car accident.

## 2. Case Presentation

A 24-year-old woman with no past history of disease or surgery was transferred to the Emergency Department of the Orthopedic Clinic of a General Hospital in Thessaloniki, Greece, after a car accident a year ago. She presented with an open fracture of the right tibia and the right humerus classified as Gustillo II [9]. Laboratory examination revealed white blood cell count 5.800/mm^3^ (75% neutrophils), hemoglobin 10.2 g/dL, platelet count 108,000/mm^3^, glucose 85 mg/dL, creatinine 0.62 mg/dL, and C-reactive protein 1.5 mg/L (normal reference < 0.5 mg/L). The patient presented no markers revealing any kind of immunocompromise (negative for human immunodeficiency virus (HIV) and hepatitis B + C, negative Mantoux test, no diabetes mellitus, and normal kidney function). Surgical debridement, open reduction, and internal fixation of the humeral fracture with 4.5 mm locking compression plate were performed. The tibial fracture was treated with intramedullary nailing. Cefoxitin, amikacin, and metronidazole were administered for 3 days. X-rays taken each month showed delayed union of the humeral fracture. One year after surgery, she was readmitted to the hospital, due to a nonunion appearing on the X-rays. The clinical examination and the inflammatory markers were normal. Surgical debridement, revision of the internal fixation (two screws were removed), and filling the nonunion site with iliac crest cancellous autograft were performed. During surgery, 3 culture specimens from superficial layers, deeper layers, and the nonunion site were taken. *R. radiobacter* was isolated only from the nonunion site, while the other two cultures came out negative. The antimicrobial susceptibility to antibiotics showed sensitivity to amikacin, ciprofloxacin, carbapenems, doxycycline, tigecycline, colistin, and co-trimoxazole (Table 1).

The patient initially was treated with intramuscular administration of amikacin for 3 weeks (500 mg/day) and then with doxycycline per os for 8 weeks (100 mg/day). She came back 3 months later showing complete remission of the infection, substantial improvement, and union on the X-ray images.

### 2.1. Orthopaedic Treatment

**Figure 1:**
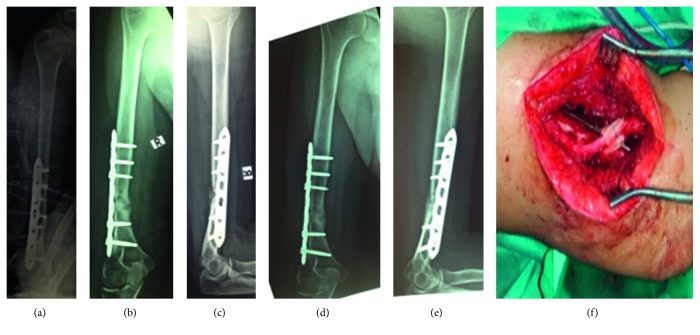
(a) X-ray after the initial treatment of the humerus fracture with a dynamic compression plate. (b and c) X-ray after the nonunion treatment. (d and e) X-ray 3 months postoperatively showing callus formation in the previous nonunion site. (f) Intraoperative picture after the nonunion treatment showing the radial nerve.

Regarding the initial fracture management, open humeral shaft fractures are commonly treated with open reduction and internal fixation using a compression plate with excellent results [10, 11]. This procedure was followed in our case and the patient had regular follow-up. During this time, the fracture failed to unite and by one year it was considered a nonunion [12]. The treatment choice of a locking compression plating and cancellous bone grafting is a reliable option in these cases [13]. The internal fixation was proven stable intraoperatively, so the revision of the osteosynthesis was relatively minimal (two screws removed), and the main surgical intervention was the nonunion fibrous tissue debridement and the filling with cancellous bone autograft harvested from the patient's iliac crest. Three months after the operation, union of the humeral shaft was achieved with a callus formation showing in the X-rays, while in other reports, the union needed more than five months in order to be achieved [14].  Figure 1 shows X-rays before and after the nonunion treatment and an intraoperative picture. The patient scored 90 points on the constant score for the shoulder evaluation, which is an excellent result, while having a full range of motion in her elbow [15].

### 2.2. Microbiological Investigation

All culture specimens were processed by Gram staining and worked out with conventional methods. Only the Gram stain from the nonunion site showed Gram-negative rods, with some appearing to have been internalized by neutrophils. Gram stains from the other two specimens were negative. Culture from the nonunion site grew a nonfermenting, Gram-negative bacillus, producing dry, tenacious colonies on blood, chocolate, and MacConkey agar, while the other two specimens were negative. The organism was identified as *R. radiobacter* using the automated system Vitek II (bioMerieux, Marcy-l'Etoile, France) and confirmed by the conventional biochemical methodology (Table 2). The antibacterial susceptibility (minimum inhibitory concentration (MIC) determination) of the isolate was performed by E-test (AB Biodisk, Solna, Sweden). Clinical Laboratory Standard Institute (CLSI) interpretive criteria for nonfermentative Gram-negative bacteria [16, 17]. The isolated *R. radiobacter* was sensitive to ciprofloxacin, amikacin, and doxycycline (Table 1).

## 3. Discussion

Although *Rhizobium radiobacter* is a pathogen commonly found in soil and plants, human disease caused by the *Rhizobium* genus is relatively rare, especially cited in immunocompromised patients and in those carrying foreign plastic bodies such as catheters. Although several case reports regarding *R. radiobacter* infections have been published during the last decade, there are no available data about the incidence of this bacterium in orthopaedic patients. In our case, the patient diagnosed with *R. radiobacter* infection was a young 24-year-old woman with normal immune function. She was not a carrier of an intravascular catheter, but she did carry foreign metal materials (internal fixation with a metal plate and an intramedullary nail) from the surgery following a car accident a year ago. It is well documented in the literature and frequently observed in clinical practice that nonunions might be caused by an indolent infection without any clinical symptoms [18].

Due to their soil habitat, *R. radiobacter* strains usually present natural resistance to several antibacterial agents. The possible explanation for this finding is their coexistence with other organisms with the ability of producing antibiotics [19]. Moreover, it is a fact that a microbe's susceptibility profile is also dependent on laboratory interpretation [19]. The CLSI has failed to determine reference breakpoints for disk diffusion susceptibility method for this bacterium, and therefore, the choice of the suitable antibiotic regimen should be best guided by MIC testing results.

According to a literature review, reported antibiotic susceptibility behaviors differ considerably among *Agrobacterium* isolates [19]. Due to low virulence and incidence, the optimal therapy for *R. radiobacter* infection has not yet been defined. Therefore, treatment of *R. radiobacter* infections should be individualised according to the antimicrobial susceptibility test [20]. In our case, the patient was initially treated with empirical antibiotic therapy based on preceding cases, and then medication was adapted to *R. radiobacter* antimicrobial susceptibility. Based on review of the existing case reports, the antibiotics usually administered for *R. radiobacter* infection treatment were aminoglycosides, third-generation cephalosporins, carbapenems, fluoroquinolones, and extended-spectrum beta-lactams [4, 6, 21]. However, resistance to gentamicin has also been reported [5]. Carbapenems and extended-spectrum beta-lactams should be kept only for cases of resistant strains in order to avoid the emergence of multiresistant isolates. Ciprofloxacin is recommended as a first-line empirical agent in treating *R. radiobacter* infections including bacteraemia [22]. Clinical evidence regarding the duration of *R. radiobacter* bloodstream infection treatment is scarce. Nevertheless, a therapy of 10 to 14 blood culture-sterile days seems to be efficient [19]. Our patient was treated with amikacin and subsequently with doxycycline and was substantially improved.

To the best of our knowledge, this is the first case of *R. radiobacter* infection in an orthopaedic young patient with an adequate immune system. Our patient did not carry catheter of any kind and probably was initially infected by this opportunistic bacterium at her car accident a year ago due to her open wound being exposed to the soil.

## 4. Conclusions

This is the first case of *R. radiobacter* infection in a nonimmunocopromised patients and the first with infected nonunion. Treatment of the nonunion site with debridement, stable bone fixation, bone grafting, targeted, combined antibiotic therapy, and close monitoring of the patient are crucial for a successful treatment.

## Figures and Tables

**Table 1:** Antibacterial susceptibility of the *R. radiobacter* strain isolated from the nonunion site.

Antibacterial agent (s)	MIC (*μ*g/ml)	Interpretation^1^
Ampicillin	32	R
Ceftazidime	128	R
Ceftriaxone	16	R
Cefotaxime	16	R
Cefepime	16	R
Amikacin	4	S
Tobramycin	>8	R
Gentamycin	>8	R
Piperacillin/tazobactam	96	R
Imipenem	0.19	S
Chloramphenicol	32	R
Vancomycin	96	R
Ciprofloxacin	2	S
Doxycycline	<4	S
Colistin	0.5	S
Tigecycline	0.5	S

^1^R: resistant; S: susceptible; I: intermediate; MIC: minimum inhibitory concentration.

**Table 2:** Biochemical characteristics of the *R. radiobacter* strain isolated from the nonunion site.

Biochemical reaction	Result
Nitrate	+
Growth in MacConkey	+
Urea hydrolysis	+
Esculin hydrolysis	+
Gelatin hydrolysis	−
ONPG (*o*-nitrophenyl-*β*-D-galactopyranoside)	+
Oxidase	+
Vogesproskauer	−
*β*-Galactosidase	−
Tryptophan deaminase	−
Ornithine decarboxylase	−
Lysine decarboxylase	−
Arginine decarboxylase	−
Motility	+
Glucose (assimilation)	+
Mannose	+
Mannitol	+
Maltose	+
Gluconate	+
Arabinose	+
Adonitol	+
Glutamyl arylamidase pNA	+
Indole	−

## References

[1] Young J. M., Kuykendall L. D., Martinez-Romero E., Kerr A., Sawada H. (2001). A revision of *Rhizobium* Frank 1889, with an emended description of the genus, and the inclusion of all species of *Agrobacterium* Conn 1942 and *Allorhizobium undicola* de Lajudie et al. 1998 as new combinations: *Rhizobium radiobacter*, *R. rhizogenes*, *R. rubi*, *R. undicola* and *R. vitis*. *International Journal of Systematic and Evolutionary Microbiology*.

[2] Young J. M., Kuykendall L. D., Martinez-Romero E., Kerr A., Sawada H. (2003). Classification and nomenclature of *Agrobacterium* and *Rhizobium*. *International Journal of Systematic and Evolutionary Microbiology*.

[3] Mastroianni A., Coronado O., Nanetti A., Manfredi R., Chiodo F. (1996). *Agrobacterium radiobacter* pneumonia in a patient with HIV infection. *European Journal of Clinical Microbiology & Infectious Diseases*.

[4] Lai C. C., Teng L. J., Hsueh P. R. (2004). Clinical and microbiological characteristics of *Rhizobium radiobacter* infections. *Clinical Infectious Diseases*.

[5] Paphitou N. I., Rolston K. V. (2003). Catheter-related bacteremia caused by *Agrobacterium radiobacter* in a cancer patient: case report and literature review. *Infection*.

[6] Plotkin G. R. (1980). *Agrobacterium radiobacter* prosthetic valve endocarditis. *Annals of Internal Medicine*.

[7] Alnor D., Frimodt-Moller N., Espersen F., Frederiksen W. (1994). Infections with the unusual human pathogens *Agrobacterium* species and *Ochrobactrum anthropi*. *Clinical Infectious Diseases*.

[8] Edmond M. B., Riddler S. A., Baxter C. M., Wicklund B. M., Pasculle A. W. (1993). *Agrobacterium radiobacter*: a recently recognized opportunistic pathogen. *Clinical Infectious Diseases*.

[9] Kim P. H., Leopold S. S. (2012). In brief: Gustilo-Anderson classification. *Clinical Orthopaedics and Related Research®*.

[10] Bell M. J., Beauchamp C. G., Kellam J. K., McMurtry R. Y. (1985). The results of plating humeral shaft fractures in patients with multiple injuries. The Sunnybrook experience. *Journal of Bone and Joint Surgery*.

[11] Harkin F. E., Large R. J. (2017). Humeral shaft fractures: union outcomes in a large cohort. *Journal of Shoulder and Elbow Surgery*.

[12] Maresca A., Sangiovanni P., Cerbasi S. (2017). Why a surgically treated humeral shaft fracture became a nonunion: review of 11 years in two trauma centers. *Musculoskeletal Surgery*.

[13] Kumar M. N., Ravindranath V. P., Ravishankar M. (2013). Outcome of locking compression plates in humeral shaft nonunions. *Indian Journal of Orthopaedics*.

[14] Abalo A., Dosseh E. D., Adabra K., Walla A., James Y. E., Dossim A. (2011). Open reduction and internal fixation of humeral non-unions: radiological and functional results. *Acta Orthopaedica Belgica*.

[15] Neumann M. V., Zwingmann J., Jaeger M., Hammer T. O., Sudkamp N. P. (2016). Non-union in upper limb fractures-clinical evaluation and treatment options. *Acta Chirurgiae Orthopaedicae et Traumatologiae Cechoslovaca*.

[16] Wayne P. (2002). *Performance Standards for Antimicrobial Disc Susceptibility Testing*.

[17] Wayne P. (2007). *Performance Standards for Antimicrobial Susceptibility Testing*.

[18] Metsemakers W. J., Kuehl R., Moriarty T. F. (2018). Infection after fracture fixation: Current surgical and microbiological concepts. *Injury*.

[19] Amaya R. A., Edwards M. S. (2003). *Agrobacterium radiobacter* bacteremia in pediatric patients: case report and review. *Pediatric Infectious Disease Journal*.

[20] Chanza M., Vidal S., Gimeno C. (2017). *Rhizobium radiobacter* in pulmonary abscess associated with postgripal necrotizing pneumonia. *Revista espanola de quimioterapia: publicacion oficial de la Sociedad Espanola de Quimioterapia*.

[21] Freney J., Gruer L. D., Bornstein N. (1985). Septicemia caused by *Agrobacterium* sp.. *Journal of Clinical Microbiology*.

[22] Chen C. Y., Hansen K. S., Hansen L. K. (2008). *Rhizobium radiobacter* as an opportunistic pathogen in central venous catheter-associated bloodstream infection: case report and review. *Journal of Hospital Infection*.

